# Child and parent behavioral and physiological reactivity to threat and distress predict children's proactive and reactive aggression

**DOI:** 10.3389/frcha.2026.1664150

**Published:** 2026-06-30

**Authors:** Kostas A. Fanti, Antonios I. Christou, Melina Nicole Kyranides, Ioannis Mavrommatis, Georgia Soursou, Nicholas D Thomson

**Affiliations:** 1 Department of Psychology, University of Cyprus, Nicosia, Cyprus; 2 Department of Special Education, University of Thessaly Volos, Greece; 3 Departments of Surgery, Psychiatry, & Psychology, Virginia Commonwealth University, Richmond, United States

**Keywords:** callous-unemotional traits, environmental sensitivity, heart rate reactivity, parent-child physiological concordance, proactive and reactive aggression

## Abstract

**Introduction:**

Threat reactivity has been linked to early manifestations of aggressive behavior, with prior research suggesting that parenting can moderate the relation between a child's psychophysiological reactivity and antisocial behavior. However, to date, few studies have examined both parents and children's bio-behavioral reactivity to emotional stimuli, and how their interaction can influence children's aggressive behavior. The present study investigated how parents' sensory processing sensitivity and heart rate (HR) reactivity to distress moderated the association between children's HR reactivity to threat, fearlessness and callous unemotional traits with reactive and proactive functions of aggression.

**Method:**

The sample consisted of 124 parent-child dyads recruited from a nationwide study on antisocial behavior.

**Results:**

Results indicate that the child's callous unemotional traits and parents' sensory processing sensitivity increase the likelihood of both reactive and proactive aggression. In contrast, the child's fearlessness mainly predicted reactive aggression. In addition, an interaction effect suggested that the child's HR reactivity to threat was negatively associated with proactive aggression when parental HR reactivity to others' distress was low, but positively associated with proactive aggression when parents' reactivity to distress was high.

**Discussion:**

Findings provide evidence for the importance of investigating physiological concordance and can inform developmental psychopathology models of antisocial behavior.

## Introduction

Childhood aggression, which is a complex set of behaviors with varying levels of biological and motivational etiology, increases the risk for psycho-social and behavioral developmental maladjustment (1–3). It is well-established that aggression can be bifurcated based on function, resulting in two subtypes called reactive and proactive aggression [e.g., (1)]. While reactive aggression includes defensive behaviors to a perceived or actual threat or provocation, proactive aggression represents well-planned and purpose-driven behaviors guided by a specific goal, such as social status or dominance [see (4)]. These two functions of aggression have a distinguishable behavioral profile, with reactive aggression associated with impulsivity and emotional dysregulation, while proactive aggression is associated with callous unemotional (CU) traits (i.e., lack of concern for others' feelings; lack of remorse or guilt; and shallow or deficient emotions), fearlessness, and reduced responsiveness to aversive stimuli [e.g., (5, 6)].

In light of these associative patterns, reactive aggression has been described as “hot-blooded,” including short-tempered, aggressive acts, while proactive aggression as “cold-blooded,” including planned harmful acts (1, 7). Although based on this distinction, reactive aggression is expected to be associated with hyper-arousal and proactive aggression with hypo-arousal, a recent literature review resulted in the conclusion that the two functions of aggression do not have distinct physiological profiles (1). The authors suggested that one way to explain these contradicting findings is to focus specifically on measures of fear and physiological reactivity to threat, since by definition proactive and reactive aggression are expected to relate to low or high responses to threat, respectively (1). Additionally, there is a need to account for individual differences in personality traits related to distinct physiological responses, such as CU traits. Finally, it is important to test whether environmental factors might moderate associations between children's physiological reactivity and aggressive behavior, testing models of biological vulnerability to environmental experiences (8). The current study takes this suggestion a step further by examining whether interactions between variables relating to child (i.e., fearlessness, CU traits, and physiological reactivity to threat) and parent characteristics (i.e., environmental sensitivity and physiological reactivity to others' distress) at both the behavioral and physiological level, influence the probability of the child displaying proactive or reactive aggression.

### Child traits and aggression

Certain individual traits like fearlessness and CU traits have emerged as important factors increasing the likelihood of aggression in youth. Proactive aggression has been associated with increased fearlessness, unemotionality, and low reactivity to others' distress, whereas reactive aggression has been associated with more fearful and impulsive reactions to distressing experiences [e.g., (1, 6, 9–11); Helfritz & Stanford, 2006]. Additionally, although some work suggests that CU traits are associated with both proactive and reactive forms of aggression (12), the majority of prior work indicates that these traits are more strongly associated with proactive aggression compared to reactive aggression [e.g., (13, 14)]. In fact, high CU traits and greater reactivity during a fear induction task were related to proactive aggression, suggesting that children with CU traits might not experience threatening situations as negative (15). Thus, it is important to examine both CU traits and fearlessness to understand engagement in both forms of aggression.

### Aggression and physiological reactivity

Individual differences in physiological reactivity in response to emotional stimuli have been well-studied in relation to early manifestations of aggression (16, 17). This line of research has highlighted that psychophysiological signatures of aggression are related to individual differences in response to threatening stimuli [e.g., (18)]. The particular focus of the relevant literature is on heart rate (HR) reactivity in response to aversive stimuli in children, which is regulated by both the Parasympathetic and Sympathetic Nervous Systems (5, 19). Changes in cardiac activity are evident when the individual is presented with threatening stimuli, which activates the “flight or fight” response (1, 20). As such, the HR reactivity index is a reliable indicator of emotional reactivity [e.g., (21)].

Several studies indicate that individuals high in antisocial behavior are characterized by reduced physiological activity when processing aversive and stressful stimuli [e.g., (5, 9, 22)]. Theoretically, such low arousal levels represent an unpleasant physiological state, and to change this negative feeling, some individuals might engage in thrilling antisocial behaviors to increase their arousal levels (23, 24). However, other work has also documented heightened reactivity in response to aversive stimuli among children displaying antisocial behavior [see the meta-analysis of (19)]. Therefore, it is likely that both hypo-arousal and hyper-arousal may be related with antisocial behavior early in life.

The distinctive and opposite patterns of the behavioral associations observed in reactive and proactive aggression may also reflect differences in physiological reactivity. Reactive aggressive behaviors relate to heightened physiological reactivity (9), highlighting that reactive aggressors are hypersensitive to fearful stimuli, while proactive aggression might be associated with hypo-arousal during fear induction [see review by (1)]. However, several studies reported opposite patterns of associations, linking proactive aggression with high HR reactivity or reactive aggression with low HR reactivity to aversive stimuli [e.g., (6, 25, 26)], while additional studies pointed to null findings (27, 28). These findings suggest that additional investigations are needed to understand physiological reactivity in relation to aggressive behavior (1).

### Aggression and parental sensitivity

Sensory Processing Sensitivity (SPS) has been widely illustrated as a significant environmental susceptibility marker that can lead to differential developmental outcomes (29, 30). Highly sensitive individuals are more prone to negative but also positive emotionality (31). From a psychophysiological perspective, high SPS has also been related with high emotional and physiological reactivity when processing emotional stimuli (29). Interestingly, mothers with high SPS experienced parenting as difficult, but at the same time, parents high in SPS exhibited more attunement to their child (32, 33). In the context of child aggression, it is likely that parents scoring high on SPS may be more aware of their child's needs but also, as a result, may become overwhelmed (by excessive information) and end up implementing ineffective parenting practices (i.e., emotionally charged parenting, inconsistent discipline). Such negative parent-child interactions might be driven by parents who are easily triggered or distressed in their parenting role, and as a result are more likely to lose their temper. Moreover, they might be more likely to report that their children are displaying elevated levels of aggression as they are less able to manage their own but also their child's behavior [e.g., (34–36)].

In addition to assessing parental sensitivity using questionnaires, the current study takes into account parents' physiological reactions to aversive stimuli. Specifically, we assessed parents' HR reactivity to others' distress in an effort to more closely resemble parents' emotional sensitivity. Although evidence is still limited, prior work suggests that parental physiological reactions are important explanatory factors for children's aggression [e.g., (37)]; however, no prior work differentiated between proactive and reactive aggression. Nevertheless, based on prior findings that parents' high autonomic reactivity is associated with lower positive parenting [see (38) for a meta-analysis], we expected that high parental physiological reactivity to distress to increase both proactive and reactive aggression.

### Interactions between child and parent individual characteristics

Familial experiences can influence aggressive behavior, and interaction models have highlighted that threat-related physiological reactivity modulates the contribution of familial variables, such as family conflict and parental psychopathology, with antisocial behavior as well as proactive and reactive aggression [e.g., (35, 39–41)]. Importantly, children exhibiting low physiological reactivity to threatening stimuli might be less emotionally sensitive to parental practices, which can be driven by their low levels of arousal, fearlessness, and lower distress (5, 42). In contrast, children characterized by high physiological reactivity may be more sensitive to negative environmental experiences, with prior work suggesting that parental distress increases the likelihood of behavioral problems only among children characterized by high physiological reactivity to threat (43). However, additional work indicated that high HR reactivity might protect children from engaging in aggressive behavior, whereas the combination of familial negativity with low physiological reactivity increased the likelihood of aggression (22). When examining subtypes of aggression, evidence has shown that baseline physiological activity was associated with increased proactive aggression under high levels of inconsistent discipline, and decreased reactive and proactive aggression under low levels of inconsistent discipline (35).

Although findings are inconsistent, they highlight the importance of testing the interaction between environmental influences and physiological activity in predicting aggression (44, 45). In addition to such interactions, we propose that it is important to test interactions between child (e.g., CU traits, fearlessness, reactivity to threat) and parental bio-behavioral characteristics (e.g., sensitivity and reactivity to distress) in relation to different forms of aggression. From a biopsychosocial perspective, such bio-behavioral exchanges may explain the underlying mechanisms that increase children's risk for aggressive behavior. In fact, parent-child physiological concordance (the degree of similarity or agreement in physiological levels between the parent and the child) has been described as an emotional sensitivity mechanism that can influence children's socio-emotional development (46–49). Interestingly, in high-risk family contexts, physiological concordance (i.e., high-high or low-low parent-child physiological reactivity) may result in negative parent-child interactions that can increase children's emotional problems [e.g., (49)]. Further, a recent study suggested that parent-child physiological concordance might increase the likelihood of aggressive behavior when negative parenting practices are present (37). While there is a plethora of research showing similar reactivity between parents and infants [for reviews see (50, 51)], evidence on such potential associations in childhood and how they may link to distinct functions of aggression are absent from the extant literature.

### Current study

This study aims to contribute to the growing body of evidence testing biosocial models of development by examining the main and interactive effects of child (i.e., fearlessness, CU traits, and HR reactivity to threat) and parental (environmental sensitivity and HR reactivity to distress) characteristics in relation to proactive and reactive aggression. We propose that children high on CU traits, who also present a fearless temperament, will be at higher risk of engaging in either proactive or reactive forms of aggression when their parents are highly sensitive to subtle environmental changes. Additionally, it is expected that parent-child physiological concordance to threatening or distressing emotional stimuli might explain engagement in either reactive or proactive forms of aggression. For example, a child with elevated physiological reactivity to threat might be more likely to engage in reactive aggression if they have a parent whose physiological reactivity is also excessive (both presenting hyperarousal). In contrast, lower physiological activation to negative stimuli by both the parent and the child is expected to be related to proactive aggression. These hypotheses are based on evidence suggesting that reactive aggression is often associated with heightened physiological reactivity in response to fear. In contrast, proactive aggression is mainly associated with under-arousal during fear induction [see (1)]. To this end, the present study aims to expand the field of knowledge by providing evidence of how physiological concordance explains heterogeneous aggressive behaviors. Identifying such bio-behavioral markers, as well as the potential concordance between parent and child biobehavioral factors, may be pivotal in designing targeted prevention and intervention programs.

Moreover, parental SPS was conceptualized primarily as a contextual moderator rather than as a focal risk factor for child aggression. Drawing on biological sensitivity to context frameworks, we expected that parental SPS would shape the emotional climate and responsiveness within the dyad, thereby conditioning associations between child physiological reactivity and aggressive behavior. Accordingly, moderation effects were exploratory in nature, while direct associations between parental SPS and aggression outcomes were modeled as covariates rather than as the primary focus of inference.

## Method

### Participants

The sample consisted of 124 dyads including data from children (*M*age = 9.98; *SD*age = 1.30, 48.8% females) and one of their parents (*M*age = 40.90; *SD*age = 4.85, 87.7% females). Families were recruited from a nationwide database of 16,000 elementary students. For the purposes of this nationwide study parents completed an online questionnaire battery distributed via REDCap, a secure internet-based platform (52). Informed consent was also provided online. We over-recruited families (50% of the sample) in which children presented elevated behavioral problems [scored + 1 SD above the mean on conduct problems, measured with the Strengths and Difficulties Questionnaire; (53)] to ensure that the selected participants would show enough variability on the measures under investigation. The rest of the sample was randomly selected. Out of the 161 families that were originally recruited, 37 were excluded from the analysis due to movement artifacts. Artifact detection was conducted through visual inspection of ECG signals and automated flagging of segments with excessive noise, signal dropout, or irregular R–R intervals. Participants were excluded if more than 20% of task-related data were contaminated by artifacts or if reliable heart rate estimates could not be obtained for either the neutral or emotional conditions. These criteria are consistent with prior work using similar experimental paradigms [e.g., (54)]. Excluded dyads did not differ from included participants on available demographic or behavioral measures (all ps > .10). The study received ethical approval from the National Bioethics Committee (No. 2019/73 & 2020/26) in Cyprus.

### Measures

#### Reactive and proactive aggression

To assess reactive and proactive aggression, parents completed the Reactive-Proactive Aggression Questionnaire [RPQ; (55)], which consists of 23 Likert-scale items assessing the frequency of aggression displayed by the child on a scale from 0 (never) to 2 (often). The RPQ has demonstrated high validity and good reliability in prior research (55, 56). In the current study, both the Reactive (11 items; *α* = .80) and Proactive (12 items; *α* = .83) subfactors showed good reliability.

#### Parents sensory processing sensitivity

Environmental sensitivity was assessed using a brief 12-item version of the Highly Sensitive Person (HSP) scale, a shorter form of the original 27-item scale, which has demonstrated comparable psychometric and construct validity properties (57). Parents rated each item on a 7-point Likert scale ranging from 1 (strongly disagree) to 7 (strongly agree). Example items include: “Do you seem to be aware of subtleties in your environment?”, “Do you get rattled when you have a lot to do in a short amount of time?”. Higher scores reflect higher sensitivity to environmental change. The scale presented good internal consistency in the current sample (*α* = .80).

#### Child's fearlessness

Fearlessness was assessed with the 6-item Child Fearlessness Scale (58). Parents rated each item (e.g., “S/he does not seem to be afraid of anything”) using a Likert scale ranging from 1 (Does not apply at all) to 4 (Applies well), with a higher score indexing fearlessness. Similar to prior work, the total score showed good internal consistency (*α* = .86) in our sample [e.g., (58, 59)].

#### Callous-unemotional traits

CU traits were assessed using the 24-item Inventory of Callous-Unemotional Traits [ICU; (60)]. Parents were instructed to rate how each item applies to their child (e.g., “He/she does not feel remorseful when he/she does something wrong”) using a 4-point Likert scale (0 = “not at all” to 3 = “definitely true”). A higher score indicates high levels of CU traits. The scale exhibited good internal consistency (*α* = .88), similar to prior work [e.g., (54)].

### Experimental information

#### Procedure

Families were assessed at the Developmental Psychopathology Lab, located at the University of Cyprus. When each family arrived (child and parent/guardian), they were familiarized with the laboratory's setting and were informed about the study. After obtaining informed consent from parents (for their and their child's participation), parents were guided to a separate room and were asked to answer different questionnaires while their child completed the task. Then parents were administered a similar experimental task while the researcher was with the child in an adjacent room. During the task participants were seated in front of a computer screen. They were fitted with physiological sensors and asked to limit their physical movement as much as possible. The researcher assessed baseline ratings and then administered the picture viewing task. During the task participants passively viewed different pictures in random order. After the task was completed, physiological sensors were removed, and families were informed about the study's objectives and thanked for their time.

#### Experimental materials

Participants viewed 6 neutral and 6 emotional pictures (children viewed threatening pictures and parents distressing pictures) taken from the International Affective Picture System [IAPS; (61)] and validated in prior work (62, 63). Stimuli were selected because threat- and distress-related cues engage overlapping defensive motivational systems and elicit convergent autonomic response patterns, particularly with respect to heart rate modulation and attentional orienting (64–66). The use of threat-related stimuli for children and distress-related stimuli for parents was developmentally informed: threat cues are known to evoke robust physiological responses in children, whereas distress cues have been linked to empathic responding and parent–child physiological concordance in adults (19, 49, 50, 67). Each picture was displayed for 5 s. Between the different pictures, there was an intertrial interval varying randomly from 5 to 10 s, during which the participant viewed a blank screen.

#### Apparatus

The timing of events and the presentation of emotional stimuli were controlled by an E-Prime 3.0 script (68). HR signals were collected using BIOPAC MP160 for Windows bioamplifiers and transducers, running AcqKnowledge 5.0 data acquisition software (Biopac Systems Inc., Santa Barbara, CA). HR measures were continuously monitored and visually inspected during the experiment, and all assessments were processed offline after the end of the experiment.

#### Heart rate (HR)

HR ratings were obtained using the Biopac electrocardiogram (ECG) module. To record ECG, we placed two 11-mm disposable Ag/AgCl pre-gelled electrodes on the right and left inner forearm of each participant. The ECG signal was amplified with a gain of 500, filtered using a Biopac ECG100C bioamplifier, and sampled online at 1,000 Hz. The ECG was high-pass filtered by 0.5 Hz, artifacts were corrected, and heart rate was calculated offline. Additionally, a visual artifact inspection was conducted. Sections with high proportions of artifacts were removed.

**HR reactivity** was computed by subtracting HR recordings obtained when participants viewed neutral pictures from HR obtained during the presentation of affective pictures (i.e., threat inducing pictures for children and distress inducing pictures for parents). The difference in HR ratings between the affective and neutral stimuli is considered an “index” score of physiological reactivity. The index score was created in this way because participants viewed different stimuli (neutral and emotional stimuli) and these conditions were comparable, as opposed to using the baseline condition, during which participants viewed a blank screen. This method has been successfully used in prior work [e.g., (54, 69)]. By subtracting neutral from affective stimuli, we also take into account Burt and Obradović (70) concern that difference scores from the baseline are influenced by the well-documented Law of Initial Values. Specifically, individuals with higher baseline activation will end up with smaller physiological reactivity, while those with lower baseline will score higher on the reactivity index. This dynamic is particularly important in developmental samples where baseline stability is harder to achieve.

### Plan of analysis

All the analyses were conducted in IBM SPSS 27. Initially, we tested the correlations between all assessed variables. We then used hierarchical multiple regression analyses with proactive and reactive aggression as the outcome variables. In step 1 of all analyses, the child's biological sex was entered, along with children's fearlessness and CU traits, parental Sensory Processing Sensitivity, and both parents and children's HR reactivity as predictors. In step 2, the 2-way interactions were included. All variables included in interaction analyses were standardized (z-scores). Interactions were visualized using the data visualization tool created by McCabe et al. (71). This procedure generates multiple small plots (i.e., individual plots created for each level of the moderator) and marginal effect plots (i.e., visualization of the regions of significance.

## Results

### Correlational analysis

Table 1 includes descriptive information as well as correlations. According to the findings, proactive and reactive aggression were inter-correlated, and both were positively correlated with CU traits. Additionally, proactive aggression was positively correlated with parental Sensory Processing Sensitivity (SPS), whereas reactive aggression was correlated with the child's fearlessness. CU traits were positively correlated with fearlessness and HR reactivity to threat. Parental and child HR reactivity scores were positively associated.

**Table 1:** Descriptive information and correlational analysis.

Variable	1	2	3	4	5	6	7
1. Proactive Aggression	1						
2. Reactive Aggression	.69**	1					
3. Child's fearlessness	.15	.27**	1				
4. Parental SPS	.16*	.12	-.05	1			
5. CU traits	.34**	.40**	.35**	-.09	1		
6. Child HR - threat	-.06	-.06	.03	.09	.21*	1	
7. Parent HR—distress	-.02	.02	.04	.07	.06	.25**	1
**Descriptive:**							
Mean	1.09	1.26	1.60	4.48	1.28	1.29	0.49
Standard Deviation (SD)	0.16	0.28	0.60	0.99	0.42	2.39	2.18

SPS, Sensory Processing Sensitivity; CU, Callous-unemotional; HR, Heart Rate;.

* *p* < .05.

** *p* < .01.

### Hierarchical linear regression analysis

#### Proactive aggression

**Figure 1:**
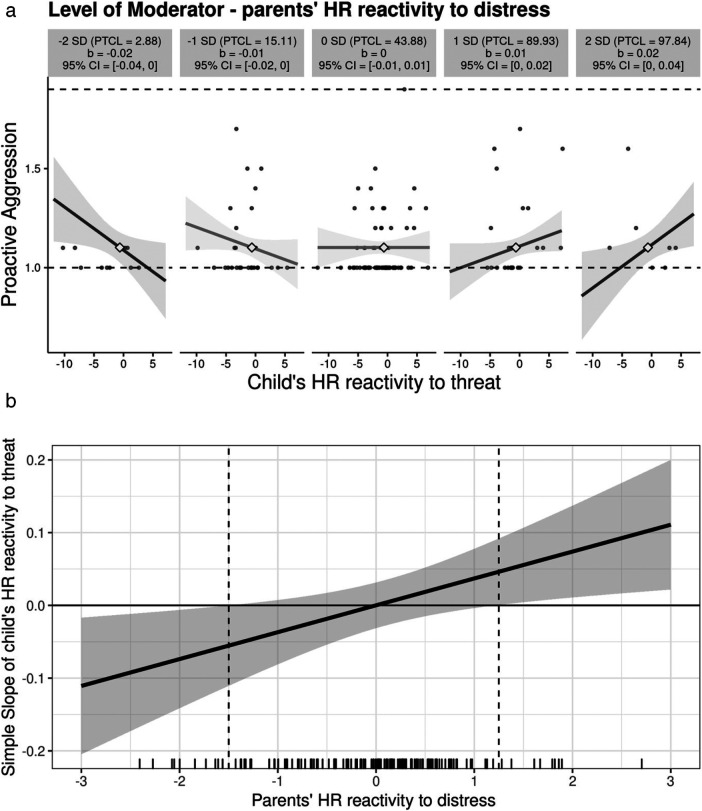
**(a)** small multiple plot of the interaction between the child's heart rate (HR) reactivity to threat with parents' reactivity to distress predicting proactive aggression. CI, confidence interval; PTCL, percentile; b, simple slope; SD, Standard deviation. **(b)** Marginal effect plot of the interaction between the child's Heart Rate (HR) reactivity to threat with parents' reactivity to distress predicting proactive aggression.

Findings from step 1 indicated that only parental SPS and CU traits were positively associated with children's proactive aggression, explaining 20% of the variance (see Table 2). As shown in step 2, the two-way interaction between the child's HR reactivity to threat and parents' reactivity to others distress was significant, explaining an additional 8% of the variance in proactive aggression. *Post hoc* probing of the interaction effect was used to determine whether the association between children's HR reactivity to threat and proactive aggression was significant at different levels of parents' HR reactivity to distress (moderator). The findings pointed to opposite associations between children's HR reactivity to threat and proactive aggression at low (−2 SD) and high (+2 SD) levels of parents' HR reactivity (see Figure 1a). Specifically, the child's HR reactivity to threat was negatively associated with proactive aggression when parental HR reactivity to others' distress was low, but positively associated with proactive aggression when parents' reactivity to distress was high. These findings also indicate that physiological concordance between child and parent physiological reactivity (i.e., low-low and high-high parent and child reactivity) places children at greater risk for proactive aggression. As shown in Figure 1b (marginal effects plot), the simple slope of the child's HR reactivity to threat on proactive aggression was significant and negative when parental HR reactivity to others' distress was −1.5 SD below the mean, with 5.68% of participants being within this region. The simple slope of the child's HR reactivity to threat on proactive aggression was significant and positive when parents' reactivity was 1.25 SD above the mean, with 6.25% of participants falling within this region. Regions of significance indicated that the interaction effects were driven by relatively small proportions of the sample at the extreme ends of the moderator distribution, and as a result these findings should be interpreted cautiously and viewed as hypothesis-generating rather than population-level effects.

**Table 2:** Results of hierarchical regression analyses.

Variable	Proactive Aggression				Reactive Aggression			
	*B*	*SE B*	*b*	*R^2^*	*B*	*SE B*	*b*	*R^2^*
**Step 1**				.20**				.28**
Child's Sex (0 = boys, 1 = girls)	.01	.05	.01		-.11	.08	-.12	
Child's fearlessness	.01	.02	.04		.06	.03	.21*	
Parental SPS	.06	.02	.30**		.07	.03	.22*	
Callous Unemotional traits	.07	.02	.38**		.11	.03	.38**	
Child HR—threat	-.02	.02	-.12		-.03	.03	-.11	
Parent HR—distress	-.01	.02	-.01		.01	.03	.01	
**Step 2**				.28**				.31**
Child's Sex (0 = boys, 1 = girls)	.01	.05	.02		-.09	.09	-.10	
Child's fearlessness	.01	.02	.07		.08	.03	.26*	
Parental SPS	.06	.02	.32**		.07	.03	.23*	
Callous Unemotional traits	.06	.02	.34**		.11	.03	.37**	
Child HR—threat	-.02	.02	-.12		-.03	.03	-.11	
Parent HR—distress	-.01	.02	-.02		-.01	.03	-.03	
HR threat * HR distress	.04	.02	.24**		.03	.03	.11	
Fearlessness * HR distress	.03	.02	.15		.03	.03	.12	
CU traits * HR distress	.02	.02	.09		.01	.04	.02	
HR threat * Parent SPS	.02	.02	.09		-.02	.03	-.05	
Fearlessness * Parent SPS	-.01	.02	-.05		.01	.03	.01	
CU traits * Parent SPS	.02	.02	.09		.01	.03	.02	

SPS, Sensory Processing Sensitivity; CU, Callous Unemotional. HR,  Heart Rate.

* *p* < .05.

** *p* < .01.

#### Reactive aggression

Findings from the regression analysis suggested that the child's CU traits and fearlessness, as well as parental SPS, were the only variables positively associated with the child's display of reactive aggression, explaining 28% of the variance. We did not identify any significant interactions in step 2, and all main effects remained significant.

## Discussion

The current study investigated how child and parental biobehavioral factors contribute to the display of proactive and reactive aggressive behavior in children. Child CU traits and parental SPS were associated with increased risk of both functions of aggression, while the child's fearless temperament was uniquely associated with reactive aggression. An interaction effect was observed when examining the physiological reactivity of parents and children to aversive stimuli. This pattern was specifically linked to proactive aggression rather than reactive aggression. Specifically, the findings indicate that higher levels of proactive aggression were evident among approximately 12% of children who showed similar direction of HR reactivity to negative stimuli as their parents (i.e., either both being highly reactive or both having low reactivity), which points to physiological concordance.

When examining child temperamental traits and their association with different forms of aggression, the current findings suggest that there are both unique and overlapping patterns. For example, CU traits were associated with increased proactive and reactive aggression, while fearlessness was only associated with reactive aggression. Indeed, CU traits were linked with both proactive and reactive forms of aggression in prior work, indicating that a constellation of characteristics associated with low empathy, unemotionality, and callousness increases the likelihood of the child engaging in distinct forms of aggressive behaviors, irrespective of the reasons leading to such behaviors (12, 56, 72). In fact, Fanti et al. (56) provided evidence that CU traits were associated with the more severe levels of co-occurring proactive and reactive aggression.

Surprisingly, the current findings suggest that the child's fearlessness, as was reported by parents, was mainly associated with reactive aggression but not proactive aggression. According to prior research and theoretical suggestions, fearlessness might be more strongly related to proactive than to reactive aggression because youth high on proactive aggression are characterized by punishment insensitivity [(23); but also see (6, 73)]. However, according to Van Til and colleagues, fearlessness can be expressed in different ways, such as engaging in risk-taking behaviors, being reckless or impulsive, and demonstrating low emotional control, which are traits associated with reactive aggression. Thus, it is likely that a fearless child may lack the inhibitory control skills that help them restrain from engaging in reactive aggression. Although the association between fearlessness and reactive aggression needs to be further investigated, our findings indicate that fearless children might respond impulsively and aggressively in response to real or perceived provocation without hesitation for the negative outcomes of their aggressive outburst, which might increase their likelihood of risk taking.

The study also unveils interesting findings regarding parents' SPS and the association with the display of aggression in their offspring/s. Prior work examining SPS demonstrated a direct link to aggression, but this was an adult sample (74). The study's results suggest that parents' SPS can also influence their child's aggressive behavior, demonstrating a link with both proactive and reactive aggression. One possibility is that parents with high SPS may be highly attuned to distress and emotional stimuli, which might make them more prone to be influenced by their child's behavior, engaging in negative parent-child interactions that increase children's antisocial behaviors. Regarding reactive aggression, SPS is associated with a disposition to being over-sensitive and over-aroused to external stimuli (29), and such parental overwhelming emotions may also destabilize the child's ability to regulate reactive aggression, particularly in situations requiring emotional restraint. When it comes to proactive aggression, one possibility is that a child may express controlling and manipulative traits, associated with proactive aggression, as a way to capitalize on their parent's emotional sensitivity in order to avoid consequences for their misbehavior. Such interpretations align with prior work showing that antisocial behavior in youth can be influenced by negative family factors like parental conflict and parenting practices [e.g. (40, 41),]. Our study introduces the possibility that the way parents process environmental change may contribute to this equation (75, 76). Due to the limited findings in this area further research is required to verify these findings.

Interestingly, the only interaction that arose as significant in the regression models was when we examined the child's and the parent's HR reactivity in response to threatening and distressing stimuli. As a result, associations between HR and proactive aggression in children identified by prior work [i.e., (77)] may be moderated by parents' physiological reactivity. Specifically, the findings indicate that when both the parent and child are attuned in their physiological reactivity (i.e., either low or excessive reactivity) to negative stimuli, the child was at greater risk for proactive aggression. Such similarity in physiological responses may reflect shared biobehavioral sensitivity profiles shaped by a combination of biological predispositions, shared environmental contexts, and reciprocal parent–child influences on emotional processing. Importantly, effective dyadic regulation typically requires a degree of physiological and emotional flexibility, with parents often expected to provide regulatory scaffolding for the child. When parent and child exhibit highly similar and inflexible physiological reactions, this may constrain adaptive regulation within the dyad, increasing the likelihood of maladaptive parent–child interactions under conditions of stress and, consequently, elevating the risk for proactive aggression as a strategy to manage emotional demands or assert control [e.g., (49)].

In contrast, parental absence of concordance, related to different extremes in physiological reactivity, protected children from engaging in proactive aggression. Although we can not draw definitive conclusions, our study indicates that a child characterized by increased reactivity to threat might need a parent who can control their emotions and who is less likely to react to others' (or their child's) increased levels of distress. On the other hand, a child with low reactivity to threat might need a parent that strongly reacts to others' emotions to balance their child's unemotionality. These important findings contradict perspectives associated with the well-known goodness of fit hypothesis. However, even though parent-child concordance is important in infancy [see the Mutual Regulation Model proposed by (78)], a recent review suggests that this concordance between the child and parent may change as the child gets older, with older children showing reduced physiological similarity (67). This could be adaptive as it is laying the foundation for future self-regulation and relying less on others to regulate one's behavior. Due to the absence of relevant evidence in the literature, this area of inquiry requires further delineation. In fact, Birk et al. (67) suggest that there are a number of factors that can affect the parent-child physiological concordance, such as parental aggression, negative parenting behaviors, and maternal depressive symptoms, which needs to be examined by future work. We should also note that the interaction effects were only significant at the extremes of the distribution, possibly suggesting that these findings only pertain to higher risk aggressive subgroups.

### Strengths, limitations, and conclusions

A key strength of the study was the integration of questionnaire-based and physiological measures, as well as the examination of parent–child physiological interactions in relation to child aggression, an area that remains underexplored. The oversampling of children with elevated behavioral problems further enhanced variability in the constructs of interest. Nevertheless, some limitations should be noted. Child aggression and temperament were assessed via parent reports, which may partly reflect parental characteristics such as sensory processing sensitivity, potentially influencing both parenting behavior and perceptions of child functioning. Although parent reports are widely used and provide valuable ecological information (79), future research incorporating multi-informant and observational approaches will be important for strengthening causal and interpretive inferences.

Heart rate reactivity in the present study indexes phasic, task-evoked changes in cardiac activity and was computed by contrasting responses to affective vs. neutral stimuli. Cardiac responses to emotionally salient stimuli involve complex temporal dynamics, typically characterized by initial orienting-related deceleration followed by mobilization-related acceleration (64, 66). Moreover, by summarizing these temporally distinct components using a subtraction-based index, the present approach limits insight into fine-grained time-course dynamics and does not disentangle sympathetic and parasympathetic contributions to autonomic regulation, which may be more precisely captured by measures such as heart rate variability (80). Accordingly, heart rate reactivity in this study should be interpreted as a relative index of task-evoked change rather than a specific marker of discrete regulatory processes. Although the sample size was sufficient to detect medium effects, replication in larger samples will be important to confirm the robustness and generalizability of the observed patterns. Future work should incorporate additional physiological measures, explicitly examine parent–child physiological synchrony using time-resolved analytic approaches, and adopt longitudinal designs to clarify developmental trajectories and causal pathways in parent–child biobehavioral dynamics.

Finally, physiological reactivity measures, including parent heart rate reactivity, exhibited substantial inter-individual variability, as reflected in relatively large standard deviations. Although such variability is common in psychophysiological data and regression diagnostics did not indicate severe violations, departures from normality cannot be ruled out. Future research should therefore consider robust or generalized linear modeling techniques better suited to skewed physiological data. Although the present sample size was sufficient to detect medium main effects, statistical power to detect interaction effects was more limited, particularly given the examination of multiple theoretically motivated interaction terms. With *N* = 124, interaction estimates may be sensitive to sampling variability, especially when effects are conditional rather than uniform across the predictor space. Model specification was guided by theory rather than *post hoc* variable selection, and no formal correction for multiple testing was applied; accordingly, findings involving interaction terms should be interpreted with appropriate caution. Replication in larger samples with preregistered analytic plans will be essential to evaluate the robustness of these interaction patterns.

Furthermore, the finding that interaction effects were most pronounced at the extremes of the moderator distribution is consistent with theoretical models emphasizing heightened sensitivity or responsivity among individuals at higher or lower ends of dispositional or physiological traits [e.g., differential susceptibility frameworks; (75)]. Because these regions represent a smaller proportion of the sample, caution is warranted in extending these effects to population-level inferences. Rather than undermining the observed associations, this pattern underscores the importance of accounting for heterogeneity in emotional and physiological responsivity and highlights the need for future studies with larger samples or targeted designs to more precisely delineate these boundary conditions. Importantly, such insights may help inform the development of more tailored intervention approaches for children exhibiting aggressive behaviors.

Additionally, the hypotheses examined in the present study do not assume equivalence of emotional stimulus content across generations. Rather, parents and children completed the same experimental paradigm, but physiological reactivity was indexed in response to different emotional conditions within that task, selected to be developmentally appropriate (distress-related cues for parents; threat-related cues for children). The hypotheses, therefore concerned between-dyad covariation in task-evoked physiological reactivity across emotional conditions, rather than stimulus-specific or time-locked physiological synchrony. Similar approaches have been used in parent–child psychophysiology research, where dyadic physiological associations are examined across different task phases or emotional contexts within a shared paradigm, and interpreted as reflecting shared sensitivity to salient affective cues rather than concurrent synchrony [e.g., (81, 82)]. Accordingly, inferences in the present study are limited to between-dyad covariation in task-evoked physiological reactivity, rather than moment-to-moment physiological synchrony or emotion-specific mirroring. Future studies should investigate child and parent physiological reactivity in response to similar stimuli, which will enable an examination of a potential mirroring effect in response to either fear or distress.

In conclusion, the present study offers critical new insights into the role of parents in shaping children's aggressive behaviors by providing some of the first evidence for an association between parent–child physiological reactivity patterns and child aggression. Specifically, the findings indicate that child–parent concordance in physiological reactivity during aversive processing is associated with the expression of proactive aggressive behavior in children. The study further highlights the potential contribution of parental environmental sensitivity to the development and manifestation of both functions of aggression. While shared physiological response patterns within parent–child dyads may reflect common biobehavioral sensitivity profiles, the present design does not permit conclusions regarding hereditary transmission or genetic mechanisms; rather, observed covariation likely arises from a combination of biological predispositions, shared environmental contexts, and parental influences on children's emotional development. As far as we know, this is among the first studies to examine the interplay between physiological responsivity and environmental sensitivity in relation to child aggression, suggesting that affective processes linked to parents may shape children's behavior above and beyond temperamental traits associated with antisocial behavior (i.e., fearlessness and callous–unemotional traits). These findings are of relevance to mental health professionals working with families to prevent aggressive behavior, underscoring the importance of considering both children's and parents' biological, behavioral, and temperamental characteristics.

## Data Availability

The raw data supporting the conclusions of this article will be made available by the authors, without undue reservation.
